# Real‐world outcomes of daratumumab, bortezomib, thalidomide and dexamethasone (Dara‐VTd) induction and lenalidomide maintenance in transplant‐eligible multiple myeloma

**DOI:** 10.1111/bjh.70775

**Published:** 2026-08-27

**Authors:** Edvan Q. Crusoé, Glaciano Ribeiro, João Tadeu D. Souto Filho, Jayr Schmidt Filho, Natalia Schutz, Milton A. F. Aranha, Abel Costa, Abrahao Hallack, Fernando V. Pericole, Juliana S. Lima, Breno Gusmão, Eduardo Flávio O. Ribeiro, Jorge P. Vaz, Rosane Bittencourt, Rafael Cunha, Danielle Ovigli, Luiza Berg, Ederson R. Mattos, Angelo Maiolino, Vania Hungria, GBRAM

**Affiliations:** ^1^ Rede D'Or Oncologia Salvador Brazil; ^2^ Universidade Federal da Bahia Salvador Brazil; ^3^ Clinica Hematológica Belo Horizonte Brazil; ^4^ Faculdade de Medicina de Campos Campos dos Goytacazes Brazil; ^5^ A.C. Camargo Cancer Center‐Rua Prof. Antônio Prudente São Paulo Brazil; ^6^ Hospital Italiano Ciudad Autónoma de Buenos Aires Argentina; ^7^ Instituto Paulista de Cancerologia—IPC São Paulo Brazil; ^8^ Instituto D'Or de Pesquisa e Ensino São Paulo Brazil; ^9^ Universidade Federal de Juiz de Fora‐Rua José Lourenço Kelmer Juiz de Fora Brazil; ^10^ Hemocentro UNICAMP Campinas Brazil; ^11^ Grupo Oncoclinicas, Instituto de Hematologia e Oncologia Curitiba Curitiba Brazil; ^12^ Beneficência Portuguesa‐Rua Martiniano de Carvalho São Paulo Brazil; ^13^ Hospital Santa Lucia Sul Brasília Brazil; ^14^ Grupo Oncoclinicas Brasilia Brasília Brazil; ^15^ Hospital de Clinicas Universidade Federal do Rio Grande do Sul‐ Rua Ramiro Barcelos Porto Alegre Brazil; ^16^ Oncoclinicas Rio de Janeiro Rio de Janeiro Brazil; ^17^ Hospital Israelita Albert Einstein São Paulo Brazil; ^18^ Fundação Cristiano Varella‐Muriaé Muriaé Brazil; ^19^ Hospital Amaral Carvalho Jaú Brazil; ^20^ Universidade Federal do Rio de Janeiro Rio de Janeiro Brazil; ^21^ Instituto Américas de Ensino Rio de Janeiro Brazil; ^22^ Department of Hematology Clínica São Germano São Paulo Brazil

**Keywords:** lenalidomide maintenance, multiple myeloma, real‐world outcomes

To the Editor,

Maintenance lenalidomide after autologous haematopoietic stem cell transplantation (AHSCT) is standard for transplant‐eligible newly diagnosed multiple myeloma (TENDMM), based on trials demonstrating an overall survival (OS) benefit following doublet‐ and triplet‐based induction with proteasome inhibitors (PIs) and immunomodulatory drugs (IMiDs).^1^, ^2^ Recently, incorporating anti‐cluster of differentiation 38 (CD38) monoclonal antibodies into first‐line induction with an IMiD and PI has established quadruplet regimens as the new standard.^3^, ^4^, ^5^ Among quadruplet regimens, PERSEUS and CASSIOPEIA represent the longest follow‐up studies to date. An indirect comparison between daratumumab with bortezomib, lenalidomide and dexamethasone (Dara‐VRd) and daratumumab‐lenalidomide (Dara‐Len) maintenance with daratumumab with bortezomib, thalidomide and dexamethasone (Dara‐VTd) and Dara/observation revealed superior efficacy for the Dara‐VRd‐based regimen, complete response (CR) (87.9% vs. 39%), minimal residual disease (MRD) negativity (75.2% vs. 64%) and progression free survival (PFS) hazard ratio (0.42 vs. 0.47), although this advantage may be confounded by maintenance differences (doublet vs. monotherapy/observation). Dara‐VRd was associated with greater grade 3/4 neutropenia (62.1% vs. 28%) and thrombocytopenia (29.1% vs. none), whereas Dara‐VTd carried thalidomide‐specific toxicities (stomatitis 13%, neuropathy).^3^, ^4^

However, optimal maintenance after anti‐CD38‐based induction remains unsettled. Previous studies of thalidomide maintenance, regardless of transplant status, failed to demonstrate an OS benefit and were consistently limited by peripheral neuropathy and venous thrombosis.^6^ Although it could be used after Dara‐VTd, thalidomide is likely to face the same limitations. Historically, lenalidomide has been the default regardless of induction, but this paradigm has been challenged by trials evaluating anti‐CD38‐based maintenance.^7^ Recently, Dara‐Len maintenance, as formally indicated in the product label, has been adopted as standard therapy after Dara‐VRd induction.^4^ Dara‐VTd significantly improved PFS and OS in the pivotal phase 3 trial, leading to regulatory approval.^3^ Notably, at the time of trial design, lenalidomide maintenance post‐AHSCT was not yet established and practice was heterogeneous; consequently, the regimen was approved without protocol‐defined maintenance. Although lenalidomide maintenance is widely used after Dara‐VTd in routine practice, no randomized study has evaluated this sequence. We therefore conducted a real‐world analysis to assess its efficacy and survival outcomes in TENDMM patients.

This study was conducted using prospectively collected data from the Brazilian Multiple Myeloma Group (GBRAM) registry and from a single centre in Argentina eligible adults (≥18 years) with newly diagnose multiple myeloma (NDMM) and AHSCT intent, enrolled from January 2018, received Dara‐VTd induction, AHSCT and two post‐transplant consolidation cycles per the CASSIOPEIA framework. However, differing from the CASSIOPEIA protocol, patients subsequently received continuous maintenance therapy with lenalidomide according to the label recommendations. As a comparative reference, we selected the Dara‐VTd arm of the CASSIOPEIA study. In that arm, half of the patients received upfront daratumumab but did not undergo daratumumab maintenance, as determined by the second randomization comparing daratumumab maintenance versus observation. Demographic, clinical and treatment data were collected. Responses were assessed according to the International Myeloma Working Group (IMWG) response criteria, including CR, very good partial response (VGPR), partial response (PR) and overall response rate (ORR) after consolidation and at the last follow‐up during the maintenance. Key time‐to‐event outcomes included OS and time to next treatment (TTNT). OS was measured from diagnosis to death from any cause. TTNT was measured from diagnosis to subsequent therapy initiation or death, whichever occurred first. OS and TTNT were estimated using the Kaplan–Meier method with pointwise 95% confidence intervals (CIs). An exploratory analysis was performed to compare OS outcomes in this cohort with those reported in the CASSIOPEIA trial population, in which daratumumab was used as maintenance therapy. Individual patient data from the CASSIOPEIA trial were reconstructed from published Kaplan–Meier survival curves using the IPDfromKM method. All analyses were performed using JAMOVI software (version 2.3), R Statistical Software (version 4.4.1) and RStudio. The study protocol was approved by the independent ethics committees at all participating sites, and written informed consent was obtained prior to data inclusion.

A total of 183 patients from 17 Brazilian centres and one in Argentina were included. Of these, 161 patients (88%) underwent AHSCT, while 22 patients (12%) did not. Reasons for not proceeding to AHSCT included refusal (*n* = 3), death (*n* = 2), progression (*n* = 3), comorbidities (*n* = 7) and unreported reasons (*n* = 7). Baseline demographic and disease characteristics were compared between the present cohort and patients from the CASSIOPEIA trial (Table 1). Differences were observed in the distribution of Eastern Cooperative Oncology Group (ECOG) performance status, with a higher proportion of patients with ECOG 0 in the Dara‐VTd‐Len cohort and a higher proportion of ECOG 1 in the CASSIOPEIA population. Regarding the International Staging System (ISS), the CASSIOPEIA group exhibited a higher frequency of ISS stage 2, while the Dara‐VTd‐Len group had a higher frequency of ISS stage 3. For the outcome analysis, patients were required to have completed the full treatment of Dara‐VTd induction, AHSCT, consolidation and lenalidomide maintenance. This landmark analysis included 137 patients. The median time from diagnosis to initiation of lenalidomide maintenance was 11 months (IQR, 10–14), and the median time from AHSCT to initiation of maintenance was 5 months (IQR, 4–7). Response rates were evaluable in 113 patients. After consolidation, 37.2% achieved a CR or better, 40.1% achieved a VGPR and 4.4% achieved a PR. At the last follow‐up, responses had deepened, with 58.4% achieving CR or better, 24.1% achieving VGPR and 4.4% achieving PR (Figure S1). After a median follow‐up of 42 months (range, 9–76), the estimated 36‐month TTNT rate was 81.1% and the 36‐month OS rate was 94.1% (Figure 1A,B). In the intention‐to‐treat (ITT) population (*n* = 183), post‐consolidation responses was 28.4% achieving ≥CR, 31.7% VGPR, 3.3% PR, and 1.6% progression disease (PD), with 35% of missing data. At last follow‐up, responses deepened to 48.1% achieving CR or better, 22.4% VGPR, 7.1% PR, and 9.3% PD, with 13.1% having unavailable data. The estimated 36‐month OS rate for ITT patients was 88.8%. It bears emphasis that CR rates are likely underestimated, as the anti‐CD38 antibody can confound immunofixation‐based response criteria. Moreover, MRD evaluation was conducted in very few patients and without standardization of assay sensitivity; consequently, we elected to exclude MRD data from the present analysis.

**TABLE 1:** Patients demographic and disease characteristics.

	DVTd + R	Cassiopeia DVTd	
	(*n* = 137)	(*n* = 543)	
Age, median (range), years	60 (35–73)	59 (22–65)	*p* = NS
Age <50 years, *n* (%)	33 (24)	83 (15)	
Sex, *n* (%)			*p* = NS
Male	74 (54)	316 (58.2)	
Female	63 (46)	227 (41.8)	
Race, *n* (%)			
White	89 (65)		
Black	19 (14)		
Not available	29 (21)		
ECOG, *n* (%)			
0	80 (58)	265 (49)	*p* = 0.01
1	29 (21)	225 (41)	*p* < 0.01
2	9 (7)	53 (10)	
3	3 (2)		
Not available	16 (12)		
Type of measurable disease, *n* (%)			
IgG	72 (53)	331 (61)	*p* = NS
IgA	33 (24)	80 (15)	*p* = 0.01
Free light chains only	24 (17)	48 (9)	*p* = 0.01
Other	8 (6)	83 (15)	
ISS, *n* (%)			
I	53 (39)	204 (38)	*p* = NS
II	35 (25)	255 (47)	*p* < 0.01
III	33 (24)	84 (15)	*p* = 0.02
Not available	16 (12)		
CRAB, total, *n* (%)	124 (91)	507 (93)	*p* = NS
Haemoglobin ≤10 g/dL	52 (38)		
Creatinine ≥2 mg/dL	17 (12)		
Bone disease	116 (85)		
Calcium >11 mg/dL	10 (7)		

Abbreviations: CRAB, calcium, renal, anaemia, bone (disease); d, dexamethasone; Dara, daratumumab; ECOG, Eastern Cooperative Oncology Group; IgA, immunoglobulin A; IgG, immunoglobulin G; ISS, International staging system; NS, not significant; T, thalidomide; V, bortezomib.

**FIGURE 1:**
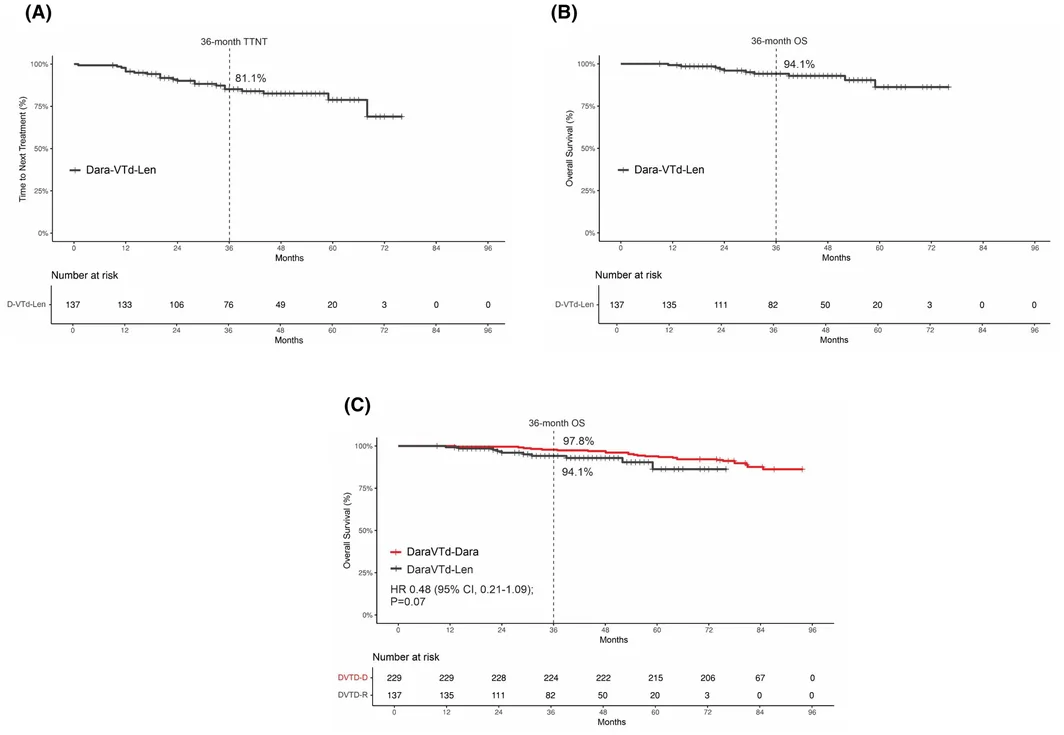
(A) Time to next treatment; (B) overall survival; (C) overall survival comparing the two groups regarding maintenance (lenalidomide vs. daratumumabe). d, dexamethasone; Dara, daratumumab; Len, lenalidomide; OS, overall survival; T, thalidomide; TE‐NDMM, transplant‐eligible newly diagnosed multiple myeloma; TTNT, Time to the next treatment; V, bortezomib.

An exploratory comparison of OS between the present cohort (Dara‐VTd‐Len) and the CASSIOPEIA trial population (Dara‐VTd‐Dara) revealed no statistically significant difference in survival outcomes (Figure 1C).

During the study follow‐up, a total of 10 patients died; six deaths were attributed to disease progression. Of the four patients who had achieved CR or VGPR, one death resulted from a second malignancy (acute myeloid leukaemia), while the remaining three by infection. Among patients with disease progression, 16 received second‐line therapy, comprising 12 distinct regimens, 8 of which included anti‐CD38 antibody combinations.

In this first real‐world assessment of sequential Dara‐VTd induction and lenalidomide maintenance in TENDMM, the regimen demonstrated feasibility and efficacy, yielding high response rates and favourable survival outcomes. Post‐transplant maintenance with lenalidomide became the standard of care after randomized studies and a subsequent meta‐analysis demonstrated a significant OS benefit.^1^ Concurrently, the induction paradigm has evolved towards quadruplet regimens incorporating anti‐CD38 monoclonal antibodies.^8^, ^9^ Studies such as CASSIOPEIA, which evaluated Dara‐VTd, consolidated the superiority of these combinations over triplets, establishing anti‐CD38‐based quadruplets as a standard of first‐line therapy. The adoption of Dara‐VTd in our setting reflects multiple factors: (1) easier access through health insurers, despite newer approvals; (2) thalidomide is freely available via the public system in Brazil; and (3) although lenalidomide is approved, its incorporation is limited by high costs and a prolonged regulatory process for oral medications. Within this rapidly evolving landscape, our analysis fills a crucial practical gap. The CASSIOPEIA trial validated Dara‐VTd induction but randomized maintenance to daratumumab versus observation, not lenalidomide—the established maintenance standard. Thus, lenalidomide after Dara‐VTd has remained an empirical, data‐limited practice. Our findings, including the deepening of responses (CR) during the maintenance phase and the 36‐month OS rate of 94.1%, validate this common practice. The exploratory indirect analysis, which compared our cohort with the daratumumab maintenance from the CASSIOPEIA study, revealed differences in baseline characteristics, particularly in ECOG and ISS stage distribution. Despite these differences, the survival curves are very similar and no significant difference in OS was observed during the analysed follow‐up period. This finding suggests that following highly effective quadruplet induction, single‐agent lenalidomide maintenance may achieve medium‐term outcomes comparable to those of parenteral monoclonal antibody therapy. It is important to emphasize that daratumumab monotherapy maintenance lacks regulatory approval, whereas lenalidomide has a well‐established position as the standard of care. The median times to initiate lenalidomide maintenance therapy from induction or after transplantation (11 and 5 months, respectively) correspond to the expected duration based on the core CASSIOPEIA protocol. The debate regarding single versus double‐agent maintenance after quadruplet induction remains open. Studies such as PERSEUS, evaluating Dara‐VRd followed by daratumumab–lenalidomide maintenance, suggest a benefit from double‐agent combination.^4^ Other studies exploring anti‐CD38‐based maintenance combinations, including isatuximab, are ongoing and still await maturation of maintenance phase data.^5^ Our findings contribute to this discussion by indicating that single‐agent lenalidomide maintenance remains an effective strategy even after potent quadruplet induction, while offering advantages in terms of convenience (oral administration) and a well‐predictable safety profile, with manageable toxicity. This study has limitations, including the non‐randomized design with inherent real‐world biases and the indirect nature of cross‐trial comparisons, which cannot replace head‐to‐head randomized evidence. The therapeutic sequence of Dara‐VTd induction followed by prolonged lenalidomide maintenance appears to be a highly effective and feasible strategy for first‐line treatment of TENDMM in clinical practice. Survival outcomes observed in this real‐world cohort were comparable to those reported with daratumumab maintenance in the pivotal CASSIOPEIA trial. This approach represents a valid therapeutic alternative, especially for patients with limited access to other quadruplet regimens or in settings where a single‐agent, well‐established oral maintenance strategy with a known toxicity profile is preferable. This study provides robust real‐world evidence supporting already widespread clinical practice and guides therapeutic decisions while we await results of direct comparative studies to define the optimal maintenance strategy in the era of anti‐CD38‐based quadruplet induction therapies.

## AUTHOR CONTRIBUTIONS

**Edvan Q. Crusoé:** Conceptualization; investigation; writing – original draft; writing – review and editing; validation; methodology; data curation; supervision; project administration; visualization. **Glaciano Ribeiro:** Investigation; methodology; formal analysis; data curation; writing – review and editing; validation. **Eduardo Flávio O. Ribeiro:** Investigation; validation; writing – review and editing. **João Tadeu D. Souto Filho:** Investigation; methodology; formal analysis; data curation; writing – review and editing; validation. **Juliana S. Lima:** Investigation; validation; writing – review and editing. **Breno Gusmão:** Investigation; validation; writing – review and editing. **Jorge P. Vaz:** Investigation; validation; writing – review and editing. **Rafael Cunha:** Investigation; validation; writing – review and editing. **Natalia Schutz:** Investigation; validation; writing – review and editing. **Abrahao Hallack:** Investigation; validation; writing – review and editing. **Milton A. F. Aranha:** Investigation; validation; writing – review and editing. **Fernando V. Pericole:** Investigation; validation; writing – review and editing. **Ederson R. Mattos:** Investigation; validation; writing – review and editing. **Danielle Ovigli:** Investigation; validation; writing – review and editing. **Jayr Schmidt Filho:** Investigation; writing – review and editing; validation. **Luiza Berg:** Investigation; validation; writing – review and editing. **Angelo Maiolino:** Investigation; validation; writing – review and editing. **Abel Costa:** Investigation; validation; writing – review and editing. **Rosane Bittencourt:** Investigation; validation; writing – review and editing. **Vania Hungria:** Investigation; writing – review and editing; visualization; validation; supervision; project administration; data curation; methodology.

## FUNDING INFORMATION

The authors have nothing to report.

## CONFLICT OF INTEREST STATEMENT

The authors have no conflicts of interest to disclose.

## Supporting information


**Figure S1.** Response rate Dara‐VTd‐Len after consolidation and last follow‐up.

## Data Availability

Data are available upon request.
