# Supplementary material for: Real‐world outcomes of daratumumab, bortezomib, thalidomide and dexamethasone (Dara‐VTd) induction and lenalidomide maintenance in transplant‐eligible multiple myeloma
Source: Br J Haematol. 2026 Aug 27;209(5):2321–5. doi: 10.1111/bjh.70775 (PMC13748401; doi:10.1111/bjh.70775)
Supplement: Supplementary file 1 [file BJH-209-2321-s001.docx]

Supplement Figure - Response rate Dara-VTd-Len after consolidation and last follow-up. PD, progression disease; PR, partial response; VGPR, very good partial response; CR, complete response.

Response Status

|  | not available | PD | PR | VGPR | CR or better |
| --- | --- | --- | --- | --- | --- |
| Response after consolidation | 17.5182% | 0.729927% | 4.37956% | 40.146% | 37.2263% |
| Response at last follow-up | 4.37956% | 8.75912% | 4.37956% | 24.0876% | 58.3942% |
